# Patient predictors of pathogenic versus commensal Gram-positive bacilli organisms isolated from blood cultures

**DOI:** 10.1017/ash.2023.506

**Published:** 2023-12-18

**Authors:** Arjun Sharma, Marion Elligsen, Nick Daneman, Philip W. Lam

**Affiliations:** 1 Department of Medicine, University of Toronto, Toronto, ON, Canada; 2 Department of Pharmacy, Sunnybrook Health Sciences Centre, Toronto, ON, Canada; 3 Division of Infectious Diseases, Sunnybrook Health Sciences Centre, Toronto, ON, Canada

## Abstract

**Objective:**

Gram-positive bacilli represent a diverse species of bacteria that range from commensal flora to pathogens implicated in severe and life-threatening infection. Following the isolation of Gram-positive bacilli from blood cultures, the time to species identification may take upward of 24 hours, leaving clinicians to conjecture whether they may represent a contaminant (inadvertent inoculation of commensal flora) or pathogenic organism. In this study, we sought to identify patient variables that could help predict the isolation of contaminant versus pathogenic Gram-positive bacilli from blood cultures.

**Design:**

Retrospective cohort study.

**Settings:**

One quaternary academic medical center affiliated with the University of Toronto.

**Patients:**

Adult inpatients were admitted to hospital over a 5-year period (May 2014 to December 2019).

**Methods:**

A total of 260 unique Gram-positive bacilli blood culture results from adult inpatients were reviewed and analyzed in both a univariable and multivariable model.

**Results:**

Malignancy (aOR 2.78, 95% CI 1.33–5.91, *p* = 0.007), point increments in the Quick Sepsis Related Organ Failure Assessment score for sepsis (aOR 2.25, 95% CI 1.50–3.47, *p* < 0.001), peptic ulcer disease (aOR 5.63, 95% CI 1.43–21.0, *p* = 0.01), and the receipt of immunosuppression prior to a blood culture draw (aOR 3.80, 95% CI 1.86–8.01, *p* < 0.001) were associated with an increased likelihood of speciating pathogenic Gram-positive bacilli from blood cultures such as *Clostridium* species and *Listeria monocytogenes*.

**Conclusion:**

Such predictors can help supplement a clinician’s assessment on determining when empirical therapy is indicated when faced with Gram-positive bacilli from blood cultures and may direct future stewardship interventions for responsible antimicrobial prescribing.

## Introduction

Gram-positive bacilli (GPB) refer to a morphologic classification of bacteria that range from commensal flora to pathogens causing severe disease.^1,2^ Due to limitations in microbiologic diagnostics, species identification of GPB can take upward of 24 hours after initial blood cultures return positive. In addition, certain Gram stain morphologies (e.g. box-car) that often can direct microbiologists toward organism identification are not readily available in initial culture reports, nor interpretable by frontline clinicians. It may therefore be difficult for clinicians to interpret initial positive blood culture results showing GPB because they may represent either a contaminant (inadvertent inoculation of blood cultures with commensal skin flora during venipuncture procedure) or a pathogen. This, in turn, may lead to an unnecessary provision of antimicrobial therapy for the former; or impact the timely initiation of antimicrobial therapy for the latter, an important component of a patient’s treatment due to the significant morbidity and mortality associated with bacteremia.^3^

It is not known whether certain aspects of a patient’s medical history or clinical presentation increase or decrease the likelihood of a GPB in the blood representing a pathogenic species (as opposed to a contaminant). By identifying patient features that predict a pathogenic species among patients with GPB in the blood, we hope to provide guidance to clinicians when faced with a positive blood culture growing GPBs.

## Methods

### Study setting and design

We conducted a retrospective cohort study of patients admitted to hospital over a 5-year period (May 2014 to December 2019) who had blood cultures reported as either *Corynebacterium* species, *Bacillus* species, *Brevibacillus* species, *Paenibacillus* species, *Clostridium* species, and *Listeria monocytogenes*. The study was conducted at Sunnybrook Health Sciences Centre, a 678-bed, academic medical center in Toronto, Ontario, Canada.

Blood cultures analyzed in this study were collected either from peripheral venipuncture or from central sources such as peripherally inserted central catheters, Hickman lines, central venous catheters (subclavian, femoral, or internal jugular), or a port-a-cath. A single blood culture set includes one anaerobic and one aerobic bottle in which a single blood collection is inoculated. Organism identification was carried out using Matrix-Assisted Laser Desorption/Ionization Time-of-Flight Mass Spectrometry.

For the purposes of this study, *Corynebacterium, Bacillus*, *Brevibacillus,* and *Paenibacillus* species were classified as GPB contaminants. In addition, to be considered as contaminants, organisms belonging to these genera must have been isolated from only a single blood culture set. In the event that a second set became positive with the same organism within 72 hours, or if the clinician requested antimicrobial susceptibility testing for the isolate, the blood culture result was no longer considered to be a contaminant and therefore excluded from this study. *Listeria monoctyogenes* and *Clostiridium* species that were isolated from blood cultures were classified as GPB pathogens.

### Data collection

A query was performed on an antimicrobial stewardship database to generate a list of patients who were 18 years of age or older at the time of blood culture collection and who met the microbiological criteria stated above. Patients with repeat blood cultures growing the same GPB from the same hospitalization were not doubly counted. Beyond identifying the GPB organism, the number of blood cultures positive, and whether other organisms were identified in the same culture, the query recorded the age, gender, and admitting service of the patient under study. Given the time of the query, the taxonomy of the organisms presented in the study may not reflect their current classification or categorization. Thus, in Table 1, where applicable, the latest taxonomy for any identified organism is provided.

**Table 1.** Species of pathogenic and contaminant Gram-positive bacilli isolated in blood cultures

Grouping	Organism	Frequency (%)
Pathogen	Clostridium species	36 (13.8)
	*C. cadaveris*	1
	*C. clostridioforme (Enterocloster clostridioformis)*	3
	*C. difficile (Clostridioides difficile)*	3
	*C. indolis (Lacrimispora indolis)*	1
	*C. innocuum*	4
	*C. paraputrificum*	3
	*C. perfringens*	7
	*C. ramosum (Thomasclavelia ramosa)*	4
	*C. septicum*	3
	*C. sordellii (Paeniclostridium sordellii)*	1
	*C. sporogenes*	2
	*C. subterminale*	3
	*C. tertium*	1
	*Listeria monocytogenes*	10 (3.8)
Contaminant	*Corynebacterium*, *Bacillus*, *Brevibacillus*, and *Paenibacillus* species	214 (82.3)

The following information was extracted from the electronic medical record for each patient identified in the database: medical conditions defined by the tenth revision of the International Classification of Diseases diagnostic codes (acute myocardial infarction, congestive heart failure, peripheral vascular disease, cerebrovascular disease, dementia, chronic obstructive pulmonary disease, rheumatologic disease, digestive ulcer, liver disease, diabetes with and without chronic complications, hemiplegia or paraplegia, renal disease, solid tumors with and without metastasis, hematologic malignancy, or human immunodeficiency virus)^4,5^; surgery or trauma within 30 days prior to blood culture draw; presence of foreign implant or device; receipt of immunosuppression within 90 days prior to blood culture draw (defined as systemic chemotherapy, corticosteroids at all doses, transplant anti-rejection mediations, or biologic agents); Quick SOFA (Sepsis Related Organ Failure Assessment) score (a scoring system for sepsis where one point is assigned for each of systolic blood pressure ≤100, respiratory rate ≥22 and GCS <15); and fever (temperature >38.0 degrees Celsius within 12 hours of culture draw).

## Analysis

A descriptive analysis was carried out for the entire study cohort on the information extracted above and then stratified based into two groups: contaminant (*Corynebacterium, Bacillus*, *Brevibacillus,* and *Paenibacillus* species) and pathogen (*Clostridium* species and *Listeria monocytogenes*). A univariable analysis was conducted comparing the variables between the two groups to identify any significant associations; Wilcoxon signed-rank and Chi-square tests were used to analyze continuous and categorical variables, respectively. The number of variables included in the multivariable model was determined using the 10:1 rule and was chosen using the backward stepwise selection method. All statistical analyses were performed using R Statistical Software.

## Results

In total, 260 unique Gram-positive bacilli blood culture results were analyzed in this study. Of these, 46 (17.7%) were identified as pathogenic organisms (Table 1). The proportion of male to female patients was similar in both groups (Table 2). Of note, 66 positive blood cultures growing a GPB belonging to the contaminant group were excluded because the organism grew in multiple sets, or the clinician requested antibiotic susceptibility testing on the isolate.

**Table 2.** Characteristics of patients with contaminant and pathogenic Gram-positive bacilli isolated in blood cultures

Variable	Contaminant Organism (*n* = 214)	Pathogenic Organism (*n* = 46)	*p*-value
Age (y, IQR)	64.00 (51.25, 79.75)	71.50 (64.00, 79.25)	0.049
Gender (% male)	129 (60.3)	28 (60.9)	1
Co-morbidities (%)			
Myocardial infarction	18 (8.4)	5 (10.9)	0.805
Malignancy	66 (30.8)	24 (52.2)	0.010
Cerebrovascular disease	52 (24.3)	5 (10.9)	0.072
Chronic obstructive pulmonary disease or other respiratory disease	17 (7.9)	2 (4.3)	0.591
Congestive heart failure	24 (11.2)	5 (10.9)	1
Dementia	18 (8.4)	2 (4.3)	0.527
Diabetes	55 (25.7)	11 (23.9)	0.947
Hemiplegia or Paraplegia	12 (5.6)	1 (2.2)	0.551
HIV	1 (0.5)	0 (0.0)	1
Liver disease	18 (8.4)	8 (15.2)	0.252
Peripheral vascular disease	17 (7.9)	3 (6.5)	0.981
Chronic kidney disease	30 (14.0)	8 (17.4)	0.721
Peptic ulcer disease	10 (4.7)	5 (10.9)	0.198
Trauma (within 30 days)	68 (31.8)	8 (17.4)	0.180
Surgery (within 30 days)	76 (35.5)	11 (23.9)	0.077
Receipt of immunosuppression	64 (29.9)	29 (58.0)	<0.001
Presence of foreign material			
Orthopedic implant	38 (17.8)	6 (13.0)	0.578
Central venous catheter	116 (54.2)	30 (65.2)	0.229
Endovascular implant	15 (7.0)	6 (13.0)	0.287
Other implant or tubes	40 (18.7)	5 (10.0)	0.290
Number of blood culture sets drawn			0.306
1	50 (23.4)	16 (35.0)	
2	143 (66.8)	24 (52.2)	
3	18 (8.4)	5 (10.9)	
4	3 (1.4)	1 (2.2)	
Number of blood culture sets positive			<0.001
1 of 4	3 (1.4)	1 (2.2)	
1 of 3	18 (8.4)	3 (6.5)	
1 of 2	142 (66.4)	13 (28.3)	
1 of 1	51 (23.8)	16 (34.8)	
2 of 3	0*	2 (4.3)	
2 of 2	0*	11 (23.9)	
Polymicrobial blood culture	48 (22.4)	16 (34.8)	0.115
Presence of fever (>38 C) within 12 hours of blood culture collection	116 (54.2)	21 (45.7)	0.373
Quick SOFA Score			0.038
0	62 (29.0)	6 (13.0)	
1	71 (33.2)	16 (34.8)	
2	71 (33.2)	18 (39.1)	
3	10 (4.7)	6 (13.0)	

Note. HIV, human immunodeficiency virus; IQR, interquartile range; SOFA, sepsis-related organ failure assessment. *Multiple blood culture positivity for contaminant group organisms was excluded as part of the study methodology.

In the univariable analysis, the patient’s age (71.5 vs. 64 years, *p* = 0.049) and the presence of malignancy (52.2% vs. 30.8%, *p* = 0.010) were associated with an increased likelihood of isolating a pathogenic versus a contaminant GPB. Furthermore, another patient factor associated with isolating a pathogenic organism was receiving immunosuppressive medications within 30 days prior to the blood culture draw (58.0% vs. 29.9%, *p* = <0.001).

In the multivariable analysis (Table 3), malignancy (adjusted odds ratio [aOR] 2.78, 95% CI 1.33–5.91), single point increments in the Quick SOFA score (aOR 2.25, 95% CI 1.50–3.47), receiving immunosuppression within 30 days of blood culture draw (aOR 3.80, 95% CI, 1.86–8.01), and a history of peptic ulcer disease (aOR 5.63, 95% CI 1.43–21.0) were associated with an increased odds of speciating a pathogenic GPB. Conversely, a diagnosis of cerebrovascular disease (aOR 0.29, 95% CI 0.09–0.76) conferred lower odds of speciating a pathogenic GPB.

**Table 3.** Multivariable analysis examining predictors of a pathogenic versus contaminant species when Gram-positive bacilli are isolated in blood cultures

Variable	Adjusted odds ratio	95% confidence interval	*p*-value
Malignancy	2.78	1.33–5.91	0.007
Quick SOFA Score (per 1 point increment)	2.25	1.50–3.47	<0.001
Receipt of immunosuppression	3.80	1.86–8.01	<0.001
Cerebrovascular disease	0.29	0.09–0.76	0.02
Peptic ulcer disease	5.63	1.43–21.0	0.01

## Discussion

Our study aimed to elucidate if certain patient factors would help predict whether a Gram-positive bacillus isolated in a positive blood culture is ultimately identified as an organism considered a pathogen versus a contaminant. Several patient variables supported this hypothesis. Malignancy, increasing Quick SOFA scores, the receipt of immunosuppressive medications, and a history of peptic ulcer disease all indicate a higher likelihood of isolating a species of pathogenic Gram-positive bacilli in the form of *Clostridium* or *Listeria monocytogenes*.

The existing body of literature supports findings from our study. For speciating a pathogenic GPB, the receipt of immunosuppression in the period prior to a blood culture draw reflects an adjusted odds ratio of 3.80 (95% CI 1.86–8.01). Immunosuppression has been associated with an increased risk of *Clostridium* and *Listeria monocytogenes* bacteremia.^6–8^ Patients may be predisposed through several mechanisms. Beyond dampening the robustness of an immune response, gut mucositis is a common complication of chemotherapy^9^ and mucosal ulceration is a known side effect of prolonged corticosteroid therapy.

As noted above, ulceration of the epithelium in the upper gastrointestinal tract may account for direct translocation of microbiota from the gut lumen into the bloodstream. However, peptic ulcer disease may also increase the risk of a pathogenic GPB bacteremia by way of proton pump inhibitor (PPI) use. Gillespie *et al*. studied the epidemiology of listeriosis in England and Wales between 2001 and 2007 and found that the use of acid-suppressing therapies was associated with bacteremias and, moreover, that bacteremic infections with *Listeria monocytogenes* over that period followed the same upward trajectory as did the prescribing patterns of PPIs.^10^ While the mechanism remains poorly understood, it is widely accepted that the gastrointestinal tract is a primary reservoir for opportunistic bacteria, and several studies have linked PPIs and states of reduced gastric acid secretion with luminal bacterial overgrowth.^11–15^

Patients who are older are more prone to develop infections.^16^ Evidence by Martin *et al.* ^17^ suggests that those above the age of 60 have a 13-fold higher relative risk of infection, and bacteremia accounts for almost 7% of infections in older adults.^18^ Factors such as comorbid illnesses, immunosenescence, nutritional deficiency, recent instrumentation, and frequent institutionalization place this patient population at an increased risk of bacteremia.^19^ Although age was not significant in our multivariable analysis, increasing with age is the risk of malignancy, which we found would predict isolating a pathogenic GPB. Interestingly, studies by Justesen *et al.* ^20^ and Kwong *et al.* ^21^ found an association between the denovo diagnosis of colonic neoplasms following the presence of *Clostridial* species in blood cultures.

Furthermore, a study by Routsi *et al.* ^22^ examined whether a SOFA score on the day of admission to the intensive care unit could predict the occurrence of a subsequent bacteremia. Their analysis of over 185 patients found that SOFA scores were independently associated with the occurrence of bacteremia (OR = 1.20, 95% CI: 1.11–1.26, *p* < 0.001), with admission scores being higher in those who developed bacteremia compared to those who did not (8.7 ± 2.7 vs 6.9 ± 3.3, *p* < 0.001). Together, with several other studies^23–25^ that support the notion that higher scores on validated organ failure assessment tools are associated with a higher mortality in patients with bloodstream infections, these findings mirror our study in that every incremental point in a patient’s Quick SOFA score was found to increase the likelihood of isolating a pathogenic GPB from a blood culture. The clinical relevance of objective measurements, such as age and Quick SOFA scores, is extended even greater importance to the outcomes in elderly patients as their clinical presentations of bloodstream infections are very often atypical.^26,27^

Though a phenomenon of post-stroke infection is documented,^28^ and is thought to result from dysregulation of the sympathetic nervous system resulting in altered gut permeability and bacterial translocation into sterile tissues in mouse models, it is unclear to us why the presence of cerebrovascular disease increased the likelihood of blood cultures returning positive for contaminant GPBs.

What distinguishes our studies from others is that it is among the first to characterize patient variables that are associated with the isolation of certain pathogenic Gram-positive bacilli in blood cultures. Furthermore, the study sample was analyzed across a large spectrum of co-morbidity, and over a prolonged timeframe prior to blood culture draw, allowing for the detection of various factors that would contribute to what many clinicians consider an indolent disease course.

Our study had several limitations. The single-center nature of this study limits its generalizability to other institutions where venipuncture (which will affect rates of blood culture contamination) practices differ. We also suspect that these results will have some limits to generalizability in highly immunocompromised populations, where some typically nonpathogenic bacteria may be associated with true infection. Other variables associated with pathogenic GPB bacteremia (such as more subjective elements of the clinical presentation and nuanced morphology of the Gram stain) were not collected during the study; and due to limitations in sample size and outcome frequency, not all confounding variables may have been accounted for in the multivariable analysis. Furthermore, contaminant organisms that were isolated in multiple blood culture sets, or for whom antimicrobial susceptibilities were requested, were excluded. As a result, this study is unable to characterize patient factors that may predispose patients to developing a bloodstream infection due to organisms typically regarded as contaminants. Lastly, as pathogenic GPBs were analyzed collectively, the nuance of clinical variables that lead to the identification of one specific organism over another may not have been captured.

## Conclusion

This study identifies patient variables that strongly predict the speciation of certain pathogenic Gram-positive bacilli following Gram stain, and can help guide clinicians in determining whether empirical therapy is warranted while final blood culture results are pending. Furthermore, findings from this study may help identify future stewardship interventions should inappropriate empiric antibiotic prescribing be identified in this patient population, meriting a focus of future research.

## Data Availability

The dataset—while not made available to any clinical data repository—can be requested through contact with the corresponding author.
